# Therapeutics in Diphtheria

**Published:** 1893-11-18

**Authors:** Benjamin Ward Richardson


### THERAPEUTICS IN DIPHTHERIA.

By Sir Benjamin Ward Richardson, M.D., F.R.S.

In the early days of practice by practitioners who are of the same age as myself, the disease called diphtheria, first fully described by Bretonneau, was unrecognised in this country. The first time my own attention was called to it was in the early part of the year 1857, when Mr. Reid, of Canterbury, sent up for the local reports of disease, published under my editorship of the *Sanitary Review*, an account of seven cases of diphtheraitic inflammation of the fauces and tonsils that had come at that time under his observation, one case proving fatal. Later on in the same year the disease began to be recognised in various parts of the kingdom. In November, 1857, Dr. Rigby, then in the tide of practice, sent a report for the weekly return of the Board of Health, in which he reported a well-marked case of diphtheria in a lady who had come up to town from Brighton. About the same time Mr. Charles P. James, a practitioner, at Leiston, in Suffolk, reported several other cases, under the title "The Epidemic Diphtherite." He was of opinion that the disease was confined to children. He had twenty cases in his practice.

This date supplies the origin of a great epidemic of the disease which extended itself rapidly in 1859, and acquired all the importance which has since been attached to it. I do not for a moment suppose that this was the first outbreak. It is certain that the affection has at times been rife in England for centuries past; but I name the first conspicuous outbreak in our own time in order that I may discuss briefly one or two points bearing on the therapeutics of the subject, following on the line of my own direct observation, as opportunities, continued up to the present time, have, I am sorry to say, too frequently occurred. Candidly, I may state at once that in the thirty-six years that have passed, it does not seem to me that any really great advance in treatment has taken place. In 1864 the disease broke out in my own family, and one most dear to me died from it. As a result I have specially kept watch over treatment in all its varied developments. I have watched by the bedside, I have read critically the records of other practitioners, but have learned little better how to proceed than when the difficulties first came before me. Mr. James, prescribing in 1857, said that the treatment he had found most effectual was, at the onset, an emetic—fifteen grains of powdered ipecacuanha—followed by small doses of dilute sulphuric acid in water, and the application to the throat, as a swab or a gargle, of a solution composed of Sir William Burnett's zinc lotion, half a fluid ounce in sixteen ounces of pure water. This treatment conveyed the idea of three elements of practice: (1) An emetic. (2) A continued remedy. (3) A local application.

*An Emetic.*—I have no doubt that in the very early stage of the disease an emetic, as suggested by Mr. James, is most useful. There is a great advantage in relieving the stomach of all contained matter in its cavity. The emetic does no harm, and food and other medicines act more promptly and effectively when its action is over.

*A Continued Remedy.*—The remedies I have seen used in the treatment of diphtheria have been chiefly quinine, iron (perchloride), and ammonia. Of the first I have little to say as to its value—in fact, I am doubtful of it. Of the second I have also doubt, and I am quite sure that in very large doses I have seen it injurious. In young children the dose should not exceed four to five minims of the tincture. Of the last remedy, ammonia, I speak with more confidence, and so convinced am I of its value that for years past I have relied on it exclusively. The late Mr. H. J. Swann, of Barrowden, in an epidemic very widespread in which he was engaged, treated every case with ammonia, with the result of 48 recoveries in 50 cases. My own results would not equal his, probably because I have not had the opportunity of treating from the onset, but they have been very good. The mode of administration which I adopt is to give the ammonia in warm milk that has previously been boiled. To half a pint of such milk I add four grains of ammonium carbonate, and have it gently warmed to new milk temperature, sweetening with an agreeable quantity of sugar, and diluted with a little water if required. Of this the patient takes *ad libitum*; it becomes a mixture that is both food and medicine. In the case of adolescents and adults I order that coffee may, if liked, be added to the milk. Ammonia has some of the best advantages. It is a good antiseptic, and, as an alkaline solvent, it has the effect of holding the blood in the fluid state, and of preventing that common fatal accident of the disease, deposition of fibrine in the cavities of the heart, and the not uncommon effect of pectous change in nervous matter, with nervous resistance and paralysis.

*Local Applications.*—From the first I have used as a local application to the membranous throat of diphtheria solution of peroxide of hydrogen of ten volume strength. I used it too timidly originally, and not so conveniently as I do now, so that I did not see its best action, and for years I alone employed it. Of late it has come into very wide service, and in America it is all but universal. For the application of the solution to the membranous surface, various modes have been devised in the form of sprays and brushes. After many trials, I have found that there is no plan so simple, practical, and effective as that of a glass pipette with an indiarubber cap, like that which is used for filling a stylographic pen. The glass must be strong, not less than five inches long, and must have a fine opening at the outlet. It must be nearly filled with the solution, and introduced into the mouth, must be made to discharge *guttatim* upon, into, and, if it can easily be done, behind the membranous coating. According to the force used in the pressure on the indiarubber cap the solution may be applied slowly so as to saturate the membrane, or it can be delivered with some force partly in spray. It may be used repeatedly without the least danger. If the surface be very extensive, a piece of cotton wool may be twisted round the bottom of the pipette, which wool, saturated with the solution by pressure from above, may be applied with a new charge of the fluid constantly filtering through it. By this means the false membrane is oxidised and destroyed better than by any other application, coming sometimes away, during a cough, as a pultaceous mass and leaving the surface beneath in contact with the oxidising liquid from which there is no risk.

The solution of peroxide before being used should be rendered feebly alkaline by the cautious addition to it of a few minims of freshly-made solution of hydrate of soda.

